# Are New Inpatient Admissions to a Psychiatric Hospital Having the Correct Blood Tests Performed in a Timely Fashion, in Accordance With RCPsych Guidelines?

**DOI:** 10.1192/bjo.2025.10560

**Published:** 2025-06-20

**Authors:** Jack Bowman

**Affiliations:** Northamptonshire Healthcare, Northampton, United Kingdom

## Abstract

**Aims:** Severe mental illness (SMI) can have a profound effect on one’s physical health. There are proven long-term effects on morbidity, mortality and total life expectancy for those living with SMI. To address this the Royal College of Psychiatrists (RCPsych) set out a standard in the publication “Standards for Inpatient Mental Health Services” that new inpatient admissions should have routine blood tests performed within 24 hours, unless they have had a recent blood test. NHFT has also adopted this view, as they have set out the same standard in “CLPr041 – Procedure for Physical Healthcare for Patients with Severe Mental Illness”. The aim of this audit was to review whether blood tests were performed within 24 hours of admission, and whether tests performed in the admission set complied with trust recommendations for physical health monitoring.

**Methods:** Clinical records were reviewed for all inpatients to a psychiatric hospital, including three general adult wards, two older adult wards, two child and adolescent wards, a rehabilitation ward and a low-secure forensic ward. Patients who were admitted on the days of data collection were excluded, as were those who were transferred to a psychiatric intensive care bed within 48 hours, who may have later returned to a general ward. Outcome measures observed were time from admission to initial bloods being performed, and compliance of blood requests with local recommendations.

**Results:** 96 patient records were reviewed. 39% of patients had a set of bloods performed within 24 hours of admission. 16% of patients did not have a set of admission bloods performed. Overall compliance with recommended admission blood investigations was 78%. The average time for admission bloods to be performed to the nearest day was 15 days for general adult wards, 2 days for child and adolescent wards, and 3 days for older adult wards.

**Conclusion:** This audit highlighted significant delays to patients having admission bloods performed. This could be improved by setting electronic reminders and mandatory fields in the electronic patient record to prompt clinicians to perform blood tests, or document clearly in the instance of patient refusal or procedural failure. Also, there was disparity in which tests were initially being ordered by different clinicians. Better compliance could be achieved by introducing a standardised blood test order set for new admissions, which could then be added to if clinically indicated. Results and recommendations have been escalated internally, with a subsequent quality improvement project in development to improve practice.

